# Prosthesis Designs and Tuberosity Fixation Techniques in Reverse Total Shoulder Arthroplasty: Influence on Tuberosity Healing in Proximal Humerus Fractures

**DOI:** 10.3390/jcm10184146

**Published:** 2021-09-14

**Authors:** Olivia Jo, Paul Borbas, Florian Grubhofer, Eugene T. Ek, Christopher Pullen, Thomas Treseder, Lukas Ernstbrunner

**Affiliations:** 1 Department of Orthopaedic Surgery, Royal Melbourne Hospital, Parkville, Melbourne, VIC 3050, Australia; jo.olivia1310@gmail.com (O.J.); cmpullen@bigpond.com (C.P.); tomtreseder@me.com (T.T.); 2 Department of Orthopaedics, Balgrist University Hospital, University of Zurich, 8008 Zurich, Switzerland; paul.borbas@balgrist.ch (P.B.); florian.grubhofer@balgrist.ch (F.G.); 3 Melbourne Orthopaedic Group, Windsor, Melbourne, VIC 3181, Australia; eugene.ek@mog.com.au; 4 Department of Biomedical Engineering, University of Melbourne, Parkville, Melbourne, VIC 3010, Australia

**Keywords:** proximal humerus fracture, reverse total shoulder arthroplasty, prosthesis design, stem design, tuberosity healing, tuberosity fixation

## Abstract

Reverse total shoulder arthroplasty (RTSA) is increasingly used for the treatment of complex proximal humerus fractures and fracture sequelae. In 2021, half a dozen models of fracture stems are commercially available, reflecting its growing utility for fracture management. Prosthesis designs, bone grafting and tuberosity fixation techniques have evolved to allow better and more reliable fixation of tuberosities and bony ingrowth. Patients with anatomical tuberosity healing not only have an increased range of active anterior elevation and external rotation, but also experience fewer complications and longer prosthesis survival. This review provides an overview of recent evidence on basic and fracture-specific RTSA design features as well as tuberosity fixation techniques that can influence tuberosity healing.

## 1. Introduction

Proximal humerus fractures are common injuries in adults, representing the third most common fracture in patients older than 60 years of age [1]. The majority of these fractures can be treated non-operatively with a high likelihood of an acceptable clinical outcome. Fractures complicated with head split or dislocation are generally treated operatively [2,3]. The management of 3- and 4-part fractures that are significantly displaced remain an area of significant variability in management, and a source of ongoing controversy [4,5]. Evidence from a multicentre randomised controlled trial suggests equivalent outcomes from open reduction and internal fixation (ORIF) or hemiarthroplasty to non-operative management in patients without a clear indication for surgical intervention [6]. There is evidence to suggest that reverse total shoulder arthroplasty (RTSA) can provide reliable functional outcomes for these displaced 3- and 4-part fractures [2,7], or even as a salvage procedure for failed ORIF [2,8]. However, RTSA for fracture is less reliable than in the cuff deficient shoulder [9,10,11]. Despite this, there has been a progressive increase in the use of RTSA for fracture management over the last decade [12,13]. The aim of this review is to gather some of the existing literature on the different tuberosity fixation techniques and prosthetic designs of RTSA that can influence outcomes in fracture management.

## 2. The Role of RTSA in the Management of Proximal Humerus Fractures

RTSA is increasingly used for the treatment of complex proximal humerus fractures in elderly patients. Compared to hemiarthroplasty, RTSA has demonstrated better clinical outcome scores, increased tuberosity healing, and lower complication rates [14,15,16]. As such, the use of hemiarthroplasty has decreased and RTSA has increased over the last decade [17,18,19,20]. In patients older than 80 years of age with comorbidities, conservative management provided similar clinical outcomes at 12 months when compared to RTSA [21], and delayed surgical treatment with RTSA did not produce inferior outcomes. Therefore, a trial of conservative management may be appropriate in this older patient population [22]. Nevertheless, there are some fracture patterns that are associated with undesirable sequelae such as non-union, mal-union, osteonecrosis, post-traumatic arthritis and locked dislocations. 

Studies demonstrated that RTSA for the treatment of fracture sequelae significantly improved range of motion, patient satisfaction and functional scores [23,24]. A registry analysis of 5946 patients reported that the cumulative revision rate at 9 years for RTSA for fractures was 7% [19]. The use of RTSA to manage 3- and 4-part proximal humerus fractures after failed operative treatment in patients younger than 60 years of age has also produced reliable functional improvements. However, higher complication and explantation rates occurred in this group [8]. In all, good clinical outcomes are associated with RTSA as a treatment option for complex proximal humerus fractures and fracture sequelae in the elderly. 

## 3. Importance of Tuberosity Healing on Function

RTSA was originally designed to compensate for rotator cuff pathology by increasing the deltoid moment arm through the medialisation of the centre of rotation [25]. In addition, its constrained articulation prevented superior humeral subluxation and provided a stable axis of rotation. This biomechanical model led to a belief that failure of tuberosity healing would be less debilitating in RTSA than hemiarthroplasty where it was a key variable determining clinical and functional outcomes [26]. However, recent studies have highlighted that tuberosity healing, as in hemiarthroplasty, is a significant outcome variable in RTSA performed in complex proximal humerus fractures [27,28,29,30,31]. The tuberosity healing rate ranges from 37 to 90% in RTSA [32,33,34,35]. An overall tuberosity healing rate of 68% was recently described in a meta-analysis [36]. Patients with anatomical tuberosity healing not only have an increased range of active anterior elevation and external rotation, but also experience fewer complications and longer prosthesis survival [37,38,39,40]. Biomechanically, a considerable drop in the joint reaction forces on the shoulder occurs with tuberosity non-union [41]. Therefore, anatomical reduction and stable fixation of the tuberosities should be attempted during RTSA.

## 4. Implant Designs

Paul Grammont popularised reverse shoulder prosthesis in 1987 [42]. The original prosthesis had a large hemispherical glenoid component with a small cup covering less than half of the glenosphere and an inlay humeral design with an almost horizontal 155° neck-shaft angle. In this design, the adduction angle is limited, resulting in scapular notching from mechanical impingement of the humerus against the inferior scapular neck [43,44]. Another important limitation of this design is its inability to restore active internal and external rotation due to decreased moment arms of the rotator cuff remnants [45]. 

Reverse prostheses have since undergone changes to address some of the problems seen with the original design. These changes aim to create a more lateralised prosthesis. Much of the focus on this lateralisation has been on modifications to the humeral stem [45,46,47,48,49,50]. The humeral neck-shaft angle of 155° was more likely to impinge on the scapula due to the reduced adduction angle. Implants with more anatomical humeral angles of 135° and 145° produced large gains in adduction and external rotation [49], and were less prone to scapular impingement [46]. 

Decreased neck-shaft angles have been combined with an onlay proximal interface in some systems. An inlay implant sits within the metaphyseal bone at the proximal component, requiring more extensive reaming. An onlay proximal interface sits on top of the neck cut, which theoretically allows for preservation of proximal bone stock including tuberosities [51]. The onlay system results in a more lateral displacement of the humerus, which produces increased tensioning of the rotator cuff and deltoid muscle efficiency [52]. In a recent study, there was an increased adduction angle and decreased notching with the onlay design compared to an inlay design [53]. 

Lateralisation can also be achieved at the glenoid side using increased sized or eccentric glenospheres, which leads to increased humeral offset, or lateralised baseplates or bony increased offset-reversed shoulder arthroplasty (BIO-RSA), which leads to increased glenoid offset [54,55,56]. In non-fracture settings, these designs have been shown to compensate for problems seen with the original Grammont design by significantly improving external rotation [57,58,59] and reducing notching [54,55,60,61]. However, some of these design features may be associated with increased rates of acromial/scapular stress fracture [60] and glenoid loosening [62]. The influence of lateralisation at the glenoid side in fracture management and tuberosity healing have not been described in the literature thus far. In addition, there are some inherent dangers in extrapolating the cuff arthropathy implant design literature to the fracture cohort. Glenoid medialisation and bone loss are common in cuff arthropathy, but in the vast majority of fractures, the glenoid is normal without medialisation. This is relevant to the use of implants with either glenoid and humeral lateralisation and achieving optimal implant position. 

**Figure 1:**
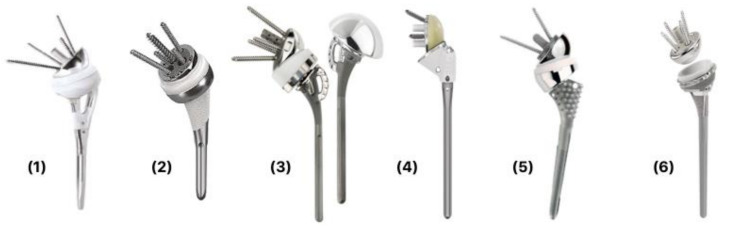
Six commercially available reverse fracture prosthesis designs: (**1**) Aequalis Reversed FX *Tonier*; (**2**) ReUnion *Stryker*; (**3**) Equinoxe Fracture *Exactech*; (**4**) Affinis Fracture *Mathys*; (**5**) Reverse Anatomical Shoulder Fracture system *Zimmer*; (**6**) Global Unite Reverse Fracture *Depuy-Synthes*.

Recently, there have been various design modifications that have been implemented to accommodate the challenges that are specific to proximal humerus fractures. Fracture stems have variable combinations of bony windows within the metaphyseal component, thus allowing bone graft, hydroxyapatite or porous coating for anatomical fixation, a lateral flange for positioning of tuberosities, and medial calcar holes for suture passage (Figure 1). These innovations are intended to allow osteointegration between implant and tuberosities [31]. Depending on the bone quality and implant design, fracture stems can either be implanted in a traditional cemented technique or cementless with a press-fit technique [63]. In a recent meta-analysis looking at outcomes of fracture stems versus non-fracture stems in proximal humerus fractures, fracture stems were shown to result in significantly improved functional scores, external rotation and forward flexion as well as tuberosity healing [64].

## 5. Stem Height

The pectoralis major tendon is often used as a landmark to determine the correct height positioning of the humeral stem in hemiarthroplasty for fracture. Accurate humeral length is an important technical factor correlated with outcome [65]. However, estimation can be challenging due to metaphyseal comminution and variations in patient size. Measurement of the distance between the superior border of the pectoralis major to the highest point of the humeral head has been suggested as a reliable method to determine the correct humeral length and was found to be, on average, 5.6 ± 0.5 cm [66]. In RTSA, the optimum implant height varies with implant design. Cagle et al. investigated this relationship for inlay and onlay designs in a cadaver study. The average distance from the superior border of the pectoralis major tendon to the top of the humeral stem was found to be 4 cm with an onlay RTSA stem and 5 cm with an inlay design, respectively. The authors also noted that if medial calcar bone remained that this appropriate height correlated with the medial aspect of the implant resting on this medial calcar bone [54]. 

## 6. Influence of Implant Designs on Tuberosity Healing

Prosthetic properties of different implants have variable biomechanical and kinematic implications for tuberosity healing in RTSA. The more traditional implant designs that evolved from the original Grammont prosthesis have been utilised in managing proximal humerus fractures. In a study involving 32 patients, a primary 155° RTSA (Delta Xtend^TM^ DePuy, Warsaw, IN, USA) maintained tuberosity fixation and achieved union in 72% of the patients [67]. Similarly, Torrens et al. treated a cohort of 41 patients with proximal humerus fractures with the same prosthesis and achieved a greater tuberosity healing rate of 68% [68]. However, Cazeneuve et al., using the Delta III reverse shoulder prosthesis, found unsatisfactory radiological outcomes including scapular notching in 70% of patients with proximal humerus fractures [69]. In another study, where RTSA was offered as a salvage treatment for failed initial surgical management, more than one-third of the patients treated with the Delta III RTSA demonstrated radiological signs of humeral loosening and almost all patients showed scapular notching [8]. Scapular notching continues to be a problem with an inlay 155° RTSA. 

Fracture specific stems with a 155° humeral inclination have shown promising results in tuberosity fixation and reduced scapular notching [31,70]. Hess et al. used the Global Unite Reverse Fracture (DePuy Synthes, Warsaw, IN, USA) for the management of 3- and 4-part proximal humerus fractures in 30 patients with a mean age of 79 years. This prosthesis has a 155° humeral inclination, a number of suture holes, porous coating consisting of titanium beads for biological fixation and backside pockets for a bone graft. The tuberosity healing rate was high, at 90%. Patients also reported high subjective satisfaction and demonstrated good active forward flexion to 140°. Ten percent of patients developed scapular notching over a 1-year follow-up. [70]. Thus, the Global Unite Reverse Fracture stem may offer satisfactory clinical outcomes with reliable tuberosity union.

Another fracture dedicated stem (Aequalis Reversed fracture, Wright Medical Group Inc., Memphis, TN, USA) yielded a high tuberosity union rate similar to or beyond those seen in a standard stem (Aequalis Reversed II, Wright Medical Group Inc., Memphis, TN, USA). The fracture stem has additional features including a hydroxyapatite coating, monobloc body, bone window, low-profile metaphysis to the inlay proximal interface and a 155° neck-shaft angle seen in the conventional design. Another group conducted a comparative study with 26 patients looking at tuberosity healing and function between a conventional stem versus a fracture-specific stem. Greater tuberosity consolidation rates were equally high in both groups (82%). Scapular notching was significantly lower with fracture stem than conventional stem (27% vs. 55%). Good to excellent clinical results were seen regardless of the stem designs used [31]. Garofalo et al. used the same dedicated fracture stem in 98 patients with acute proximal humerus fractures and resulted in a radiological tuberosity union rate of 75% [30]. In comparison, Chun et al. demonstrated an anatomical tuberosity healing rate of only 37% in a cohort of 41 patients using a conventional stem [35]. The Aequalis fracture stem may be superior, or at least equally as good as, the conventional stem for tuberosity union.

The association between anatomical neck-shaft angles and tuberosity union has been reported. In a retrospective case series involving 38 patients with a mean age of 77 years, the 135° Univers Revers (Arthrex, Naples, FL, USA) was used to treat acute proximal humerus fractures. Tuberosity healing occurred in 82% of the patients and resulted in significantly increased abduction, forward flexion and external rotation compared to those with non-union. Scapular notching occurred in 8% of the cases [71]. A biomechanical study comparing reverse prosthesis with either 135° or 155°, demonstrated that tuberosity reattachments were significantly more stable in prosthesis with an anatomical humeral angle of 135° compared to 155°. Furthermore, a 135° humeral inclination allowed an exact anatomical repositioning of tuberosities while this was not possible for the 155° design [72]. A systematic review looked at the performance of neck-shaft angles of 135°, 145° and 155° in 873 patients from 21 studies. A one hundred and thirty-five-degree inclination had the highest tuberosity healing rate of 83% compared to 69% in the 145° group and 66% in the 155° group [73]. Consequently, a 135° humeral neck-shaft angle may provide a more favourable tuberosity healing rate, compared to a more horizontal 155° stem.

The onlay implant design has been shown to have a comparable tuberosity union rate to an inlay design. Grubhofer et al. [2] treated 51 patients with a mean age of 77 years for an acute, complex proximal humerus fracture with an onlay 135° RTSA (Zimmer Reverse Anatomical Shoulder System, Zimmer, Warsaw, IN, USA) using a fracture stem. The stem had fracture spikes and stem suture holes for stable anchoring of the tuberosities. An overall tuberosity healing rate of 81% was seen in the study at a 35-month follow-up. Patients with tuberosity resection or malunion had inferior clinical outcomes. Scapular notching occurred in 63% of the patients. Satisfactory tuberosity healing is seen with an onlay interface. 

## 7. Tuberosity Fixation Techniques

Reconstruction of displaced tuberosities should be attempted to enable maximal (inter) fragmentary stability. Along with implant characteristics, several fixation techniques have been developed to impart satisfactory reattachment of the tuberosities [31,74,75,76,77]. Some studies have focused on stem-based fixation of tuberosities [30,75,78] while others have explored bone grafting [32,33,79,80,81] and suture techniques [74,77,82] to secure the tuberosities to the humeral stem. Cemented humeral fixation has not improved tuberosity healing, but has led to worse patient-reported outcomes [83,84]. 

**Figure 2:**
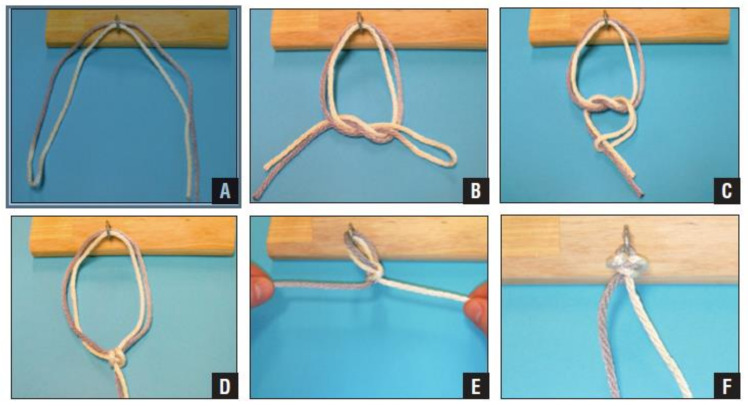
Nice Knot technique. (**A**) A double-over suture is threaded around the tissue. (**B**) A single square knot is thrown. (**C**) The two free limbs are passed through the loop. (**D**) The knot is dressed. (**E**) The knot is tightened by pulling the two free limbs apart. (**F**) Three half-hitches are applied to secure the knot. Reprinted with permission from [88]. Copyright 2017. SLACK^R^.

The most popular technique of tuberosity fixation is the use of cerclages. Double suture loop cerclage techniques have been shown to be up to three times stronger than single-stranded knots in an in vitro study. Of the 12 different knots tested, the cow hitch technique was the stiffest and strongest, followed by the Nice Knot [85]. The Double Suture Nice Knot has been suggested for fixation of the greater tuberosity after acute fracture, non-union or malunion. The technique creates a sliding knot that is self-stabilising, simple and strong (Figure 2). No studies have been performed on the reliability of the Nice Knot in greater tuberosity fixation, although it has been shown to be a safe and effective fixation method in comminuted clavicle fractures [86], and displaced patella fractures [87]. Further studies are recommended to test this fixation technique for greater tuberosity in complex proximal humerus fractures. 

**Figure 3:**
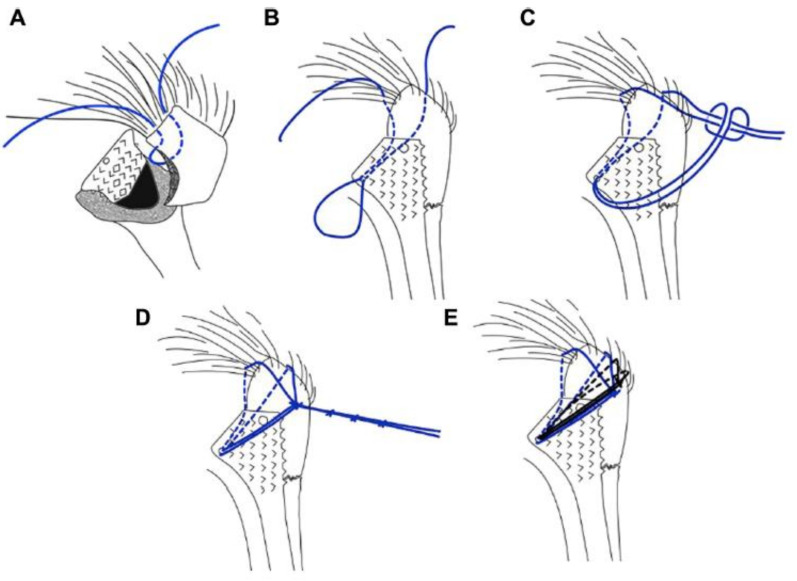
Illustration of the cow hitch technique. A suture loop is created intra-articularly at the tendon-bone interface of the in-fraspinatus (**A**). The loop is passed through the medial calcar hole of the prosthetic stem (**B**). Two loops are created by folding the loop twice (**C**) and the two free limbs are fed through the double loop (**D**), creating a self-locking mechanism. A second ‘cow hitch’ is thrown (**E**). Reprinted with permission from [74]. Copyright 2021. Elsevier.

Grubhofer et al. recently adopted a cow hitch suture technique (Figure 3) specifically for tuberosity fixation in RTSA and hemiarthroplasty in a cadaveric study [74,89]. The cow hitch suture cerclage as a stem-based suture technique using ZimmerBiomet (Warsaw, IN, USA) stems produced a significantly more stable fixation than a previously described technique by Frankle et al. [90]. The cow hitch cerclage knot utilises a self-locking mechanism, which is thought to maintain better fixation stability than the conventional knots [74,89]. The use of a tensioning device, whilst tightening the applied cerclage sutures for tuberosity fixation, has also been shown to enhance biomechanical stability and reduce rotational movement of the attached tuberosities [91]. These biomechanical results are promising and warrant further clinical studies. 

**Figure 4:**
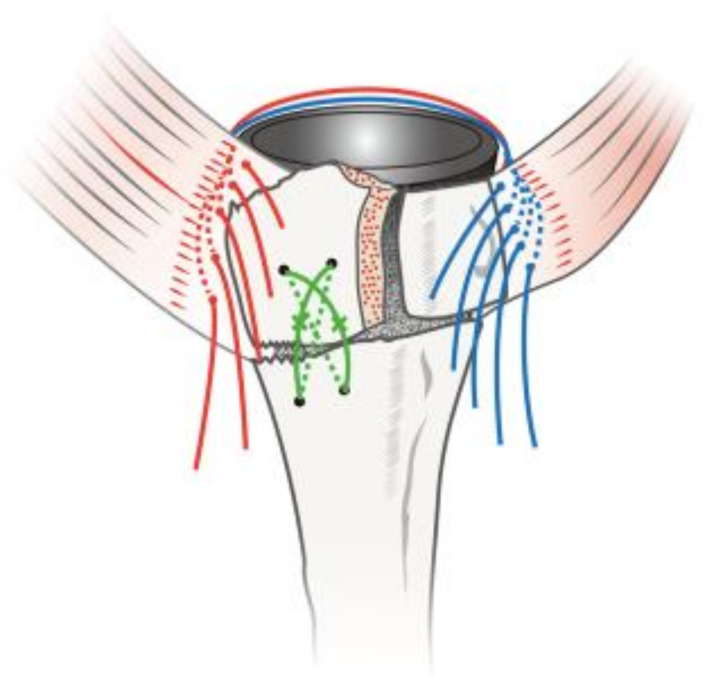
Turned suture tension band technique. Five sutures are passed through the suture hole of the humeral stem. Five sutures are turned clockwise and the other five are turned counter clockwise around the stem. Reprinted with permission from [82]. Copyright year 2021. Elsevier.

Takayama et al. also introduced a turned stem tension band technique in 18 patients with complex humerus fractures. The technique utilises ten sutures, five of which turn counter clockwise while the other five turn clockwise around the stem. Of the five counter clockwise sutures, two are applied to the teres minor muscle and three to the infraspinatus tendon. The other five clockwise sutures are applied to the subscapularis muscle (Figure 4). The technique yielded a 100% tuberosity healing rate over a 34.5-month follow-up [82]. Furthermore, Formaini et al. introduced the black and tan technique, which utilises vertical, horizontal and cerclage sutures to allow the integration of bone graft between the cement mantle and proximal extent of the humeral shaft. It yielded an 88% tuberosity healing rate in 25 patients [77]. These techniques may be simple and reproducible tuberosity fixation methods in theory, but require a period of familiarisation as they can be challenging intraoperatively. 

The use of bone graft has shown promising results in tuberosity healing in RTSA [32,33,79,80,81]. Boileau et al. [32] demonstrated, in 38 elderly patients with displaced 3- and 4-part fractures, that the Aequalis Reverse-Fracture prosthesis (Tornier, Edina, MN, USA) combined with bone grafting resulted in an 84% tuberosity union rate. The fracture stem is designed for better integration of bone graft and anatomic placement of tuberosity due to the features described earlier in this review. Uzer et al. [81] also found, in their study involving 33 patients, that cancellous block autograft augmentation led to increased tuberosity union in 77.8% of the cases versus 40% in cases treated without grafting. Functional outcomes, as well as external rotation and forward flexion, were significantly improved in the autograft augmentation group. Levy et al. [79] described a ‘horseshoe’-shaped bone graft, which led to an 86% tuberosity union rate in seven elderly patients with complex proximal humerus fractures. The authors reported that this method increased surface area for tuberosity healing and bony ingrowth. Techniques to facilitate tuberosity healing continue to evolve.

## 8. Conclusions

RTSA represents a good surgical option in complex proximal humerus fractures in the elderly population with tuberosity comminution, a head split component, fracture-dislocation and risk factors for rotator cuff pathology and non-union. It offers reliable pain relief and a good range of motion with a low revision rate. Anatomic reduction and secure tuberosity fixation with bone graft augmentation should be attempted in all cases as a good union can result in superior functional outcomes with lower complication rates. The use of a fracture-specific stem appears to improve tuberosity union rates. Suture techniques, stem-based fixation techniques, fracture-dedicated implants and the use of bone grafting and cement continue to evolve.
